# Alpinumisoflavone ameliorates choroidal neovascularisation and fibrosis in age-related macular degeneration in in vitro and in vivo models

**DOI:** 10.1038/s41598-022-18531-y

**Published:** 2022-08-22

**Authors:** Eunhye Yu, Yunjeong Song, Sun Mi Gu, Yang Hee Jo, Sang Won Yeon, Kyu Jin Han, Mi Kyeong Lee, Jung Kee Min, Jaesuk Yun

**Affiliations:** 1 College of Pharmacy, Chungbuk National University, 194-31 Osongsaengmyeong 1-ro, Osong-eup, Cheongju-si, Chungcheongbuk-do 28160 Republic of Korea; 2 College of Pharmacy, Chungbuk National University, 194-21 Osongsaengmyeong 1-ro, Osong-eup, Cheongju-si, Chungcheongbuk-do 28160 Republic of Korea; 3 Department of Ophthalmology, Ulsan University Hospital, University of Ulsan, College of Medicine, 877, Bangeojinsunhwando-ro, Dong-gu, Ulsan, 44033 Republic of Korea

**Keywords:** Drug discovery, Drug screening, Pharmaceutics, Pharmacology

## Abstract

Age-related macular degeneration (AMD) is a major cause of vision loss in the elderly population. Anti-vascular endothelial growth factor (VEGF) antibody therapy is applicable to neovascularisation of AMD; however, the prevention of fibrosis after anti-VEGF monotherapy is an unmet medical need. Subretinal fibrosis causes vision loss in neovascular age-related macular degeneration (nAMD) even with anti-VEGF therapy. We report the anti-fibrotic and anti-neovascularisation effects of alpinumisoflavone (AIF), an isoflavonoid derived from unripe *Maclura tricuspidata* fruit, in in vitro and in vivo models. For in vitro study, we treated H_2_O_2_ or THP-1 conditioned media (TCM) following activation with phorbol 12-myristate 13-acetate (PMA) and lipopolysaccharide (LPS) in a human retinal pigment epithelial cell line (ARPE-19). Choroidal neovascularisation (CNV) was induced by laser photocoagulation in mice, immediately followed by intravitreal administration of 25 μg AIF. CNV area and fibrosis were measured 7 days after laser photocoagulation. AIF showed anti-fibrosis and anti-neovascularisation effects in both the models. The laser induced CNV area was reduced upon AIF administration in nAMD mouse model. Additionally, AIF decreased the levels of the cleaved form of crystallin alpha B (CRYAB), a chaperone associated with VEGF stabilisation and fibrosis. Our results demonstrate a novel therapeutic application of AIF against neovascularisation and fibrosis in nAMD.

## Introduction

Age-related macular degeneration (AMD) is a degenerative eye disease and is the leading cause of irreversible vision loss in elderly people^1^. It is a complex multifactorial disease, and its pathogenesis is not fully understood^2^. Choroidal neovascularisation (CNV), vascular leakage, and haemorrhage are the hallmarks of nAMD^3^. Anti-vascular endothelial growth factor (VEGF) pharmaceutical products have successfully been used as a first-line medication for nAMD treatment^4–7^. However, subretinal fibrosis is a risk factor for vision loss that occurs despite anti-VEGF treatment^8^. Subretinal fibrosis may be initiated by CNV development with haemorrhage and the inflammatory response of the retinal and subretinal regions. Various cell types, such as retinal pigment epithelium (RPE) cells, immune cells, and fibroblasts, are associated with this pathological process, which consequently induces tissue scarring and blindness in patients with nAMD^9^. However, RPE cells are also known to produce VEGF, which is a target of nAMD treatment^10,11^ and express several fibrosis markers dependent on the epithelial-to-mesenchymal transition process^12^.

In contrast to anti-VEGF pharmaceutics, there are no effective anti-fibrotic agents available for nAMD treatment thus far. Therefore, the development of an anti-fibrotic agent as well as anti-neovascularisation therapy may facilitate better therapeutic intervention of nAMD.

Alpinumisoflavone (AIF) is an isoflavonoid isolated from the unripe fruits of *Maclura tricuspidata* (previously known as *Cudrania tricuspidata*)^13–16^. AIF has various pharmacological activities such as anti-inflammatory^17^, anti-metastatic^18^, and antioxidant^17^ effects. Notably, methylalpinumisoflavone shows anti-VEGF activity via hypoxia-inducible factor-1 (HIF-1) inhibition^19^ and flavones has anti-fibrotic activity^20,21^.

In this study, we tested the effects of AIF on expression level of VEGF, fibrosis markers such as alpha smooth muscle actin (alpha-SMA) and collagen type I (collagen I) in ARPE-19 cell, which has been widely used in eye research^22^, besides measuring the CNV area and fibrosis in an nAMD mouse model following AIF treatment in an attempt to elucidate whether AIF demonstrates anti-neovascularisation and anti-fibrotic activity in nAMD models. In addition, we studied the effects of AIF on the expression levels of chaperones associated with VEGF and fibrosis markers with the aim of deciphering the molecular mechanism underlying AIF activity in nAMD in vivo and in vitro models.

## Results

### TCM increased vascular endothelial growth factor A expression in ARPE-19 cells

**Figure 1:**
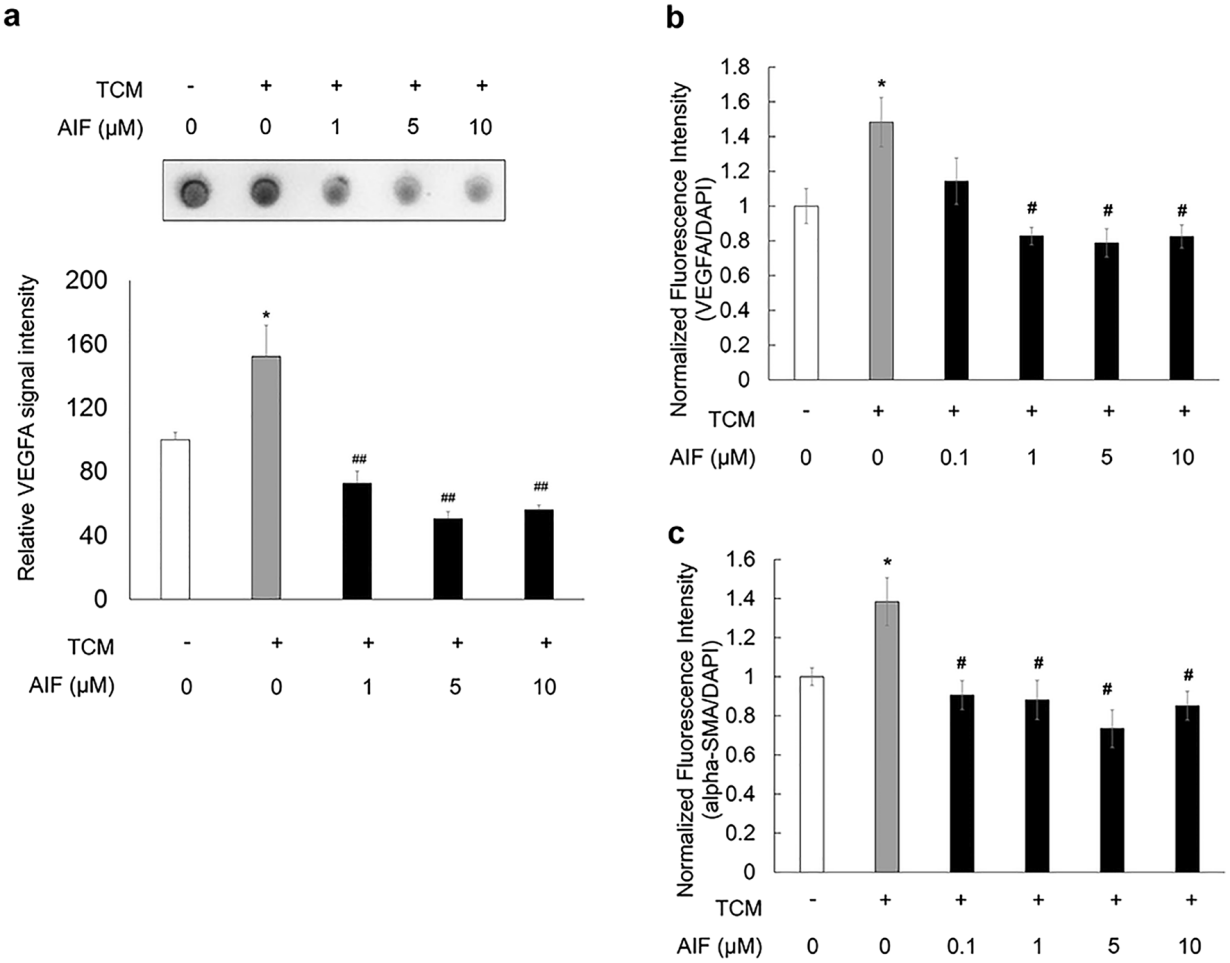
Inhibitory effects of alpinumisoflavone (AIF) on THP-1 conditioned media (TCM)-induced expression of vascular endothelial growth factor A (VEGFA) in ARPE-19 cells. (**a**) ARPE-19 cells were treated with 0.1% DMSO in RPMI-1640 complete media or AIF (0, 1, 5, or 10 µM) in TCM. VEGFA expression was measured by dot blot. The data were analysed using one-way ANOVA followed by the Holm-Šídák post-hoc t-test (*p < 0.05 vs. non-TCM group, ^##^*p* < 0.01 vs. cells treated with only TCM group) and are expressed as mean ± S.E. (n = 3–6). Uncropped blot images are presented in supplementary information (Supplementary Fig. 2). (**b**,**c**) ARPE-19 cells were treated with 0.1% DMSO in RPMI-1640 complete media or AIF (0, 0.1, 1, 5, or 10 µM) in TCM. The fluorescence intensity was measured using the FlexStation 3 Multi-Mode Microplate Reader. Data were analysed using one-way ANOVA followed by the Holm-Šídák post-hoc t-test test (*p < 0.05 vs. non-TCM group, #p < 0.05 vs. cells treated with only TCM group) and are expressed as the mean ± S.E. (n = 6–9).

Macrophages play a critical role in the development of AMD^23–25^. Thus, we stimulated ARPE-19 cells with TCM to induce macrophage inflammatory responses, so that we can evaluate the effect of TCM on vascular endothelial growth factor A (VEGFA) expression after 3 h of treatment in ARPE-19 cells. The expression level of VEGFA in ARPE-19 cells was increased by TCM. However, AIF co-treatment inhibited VEGFA expression induced by TCM. The most significant reduction of VEGFA was shown in AIF of 5 µM (Fig. 1a; *F*(4,16) = 9.627, *p* < 0.001).

We performed immunocytochemical analysis to determine the expression of VEGFA and alpha-SMA in ARPE-19 cells. The fluorescence intensity of VEGFA or alpha-SMA was increased by TCM. We found that 1, 5, and 10 μM AIF co-treatment inhibited fluorescence intensity of VEGFA and 0.1, 1, 5, and 10 μM AIF co-treatment inhibited fluorescence intensity of alpha-SMA expression induced by TCM (Fig. 1b; *F*(5,36) = 6.412, *p* < 0.001; Fig. 1c; *F*(5,36) = 6.804, *p* < 0.001).

### H_2_O_2_ increased VEGFA expression in ARPE-19 cells

**Figure 2:**
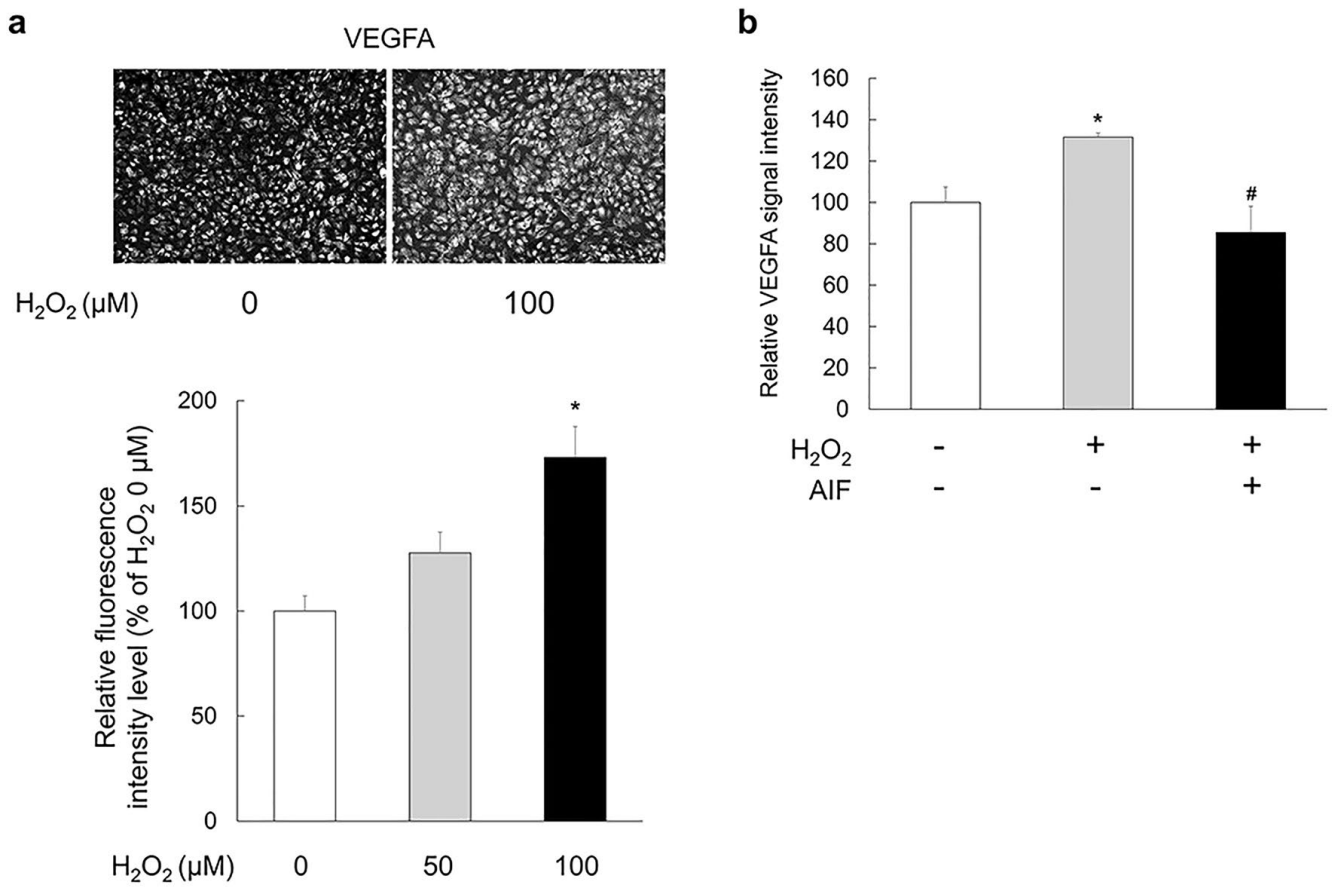
Inhibitory effects of alpinumisoflavone (AIF) on H_2_O_2_-induced expression of vascular endothelial growth factor A (VEGFA) in ARPE-19 cells. (**a**) ARPE-19 cells were treated with the indicated concentrations of H_2_O_2_ for 24 h. The fluorescence intensity of ARPE-19 cells treated with H_2_O_2_ were measured. Data were analysed using one-way ANOVA followed by the Holm-Šídák *post-hoc* t-test (*p < 0.05 vs. H_2_O_2_ 0 µM group) and are expressed as mean ± S.E. (n = 328–406). (**b**) VEGFA expression was measured by dot blot. ARPE-19 cells stimulated with H_2_O_2_ were treated with 5 µM AIF. Data were analysed using one-way ANOVA followed by the Holm-Šídák *post-hoc* t-test (*p < 0.05 vs. non-H_2_O_2_ group, ^#^*p* < 0.05 vs. cells treated with only H_2_O_2_ group) and are expressed as mean ± S.E. (n = 3–9).

It was known that oxidative stress is associated with VEGF expression^26–30^. Therefore, we also evaluated the effect of H_2_O_2_ on VEGF expression after 24 h of treatment in ARPE-19 cells. VEGFA expression increased after the addition of 50 and 100 µM H_2_O_2_ to ARPE-19 cells. We found that ARPE-19 cells treated with 100 μM H_2_O_2_ for 24 h significantly increases fluorescence intensity of VEGFA (Fig. 2a; *F*(2,1093) = 11.429, *p* < 0.001).

### AIF reduced the expression level of VEGFA in ARPE-19 cell lysates

ARPE-19 cells were treated with 5 µM AIF for 24 h under low oxidative stress conditions (100 µM of H_2_O_2_). Thereafter, dot blot analysis was performed to detect VEGFA expression. VEGFA expression decreased under AIF treatment conditions (Fig. 2b; *F*(2,13) = 5.469, *p* = 0.019).

### AIF reduced RNA expression level of alpha-SMA and collagen I in ARPE-19 cell lysates

**Figure 3:**
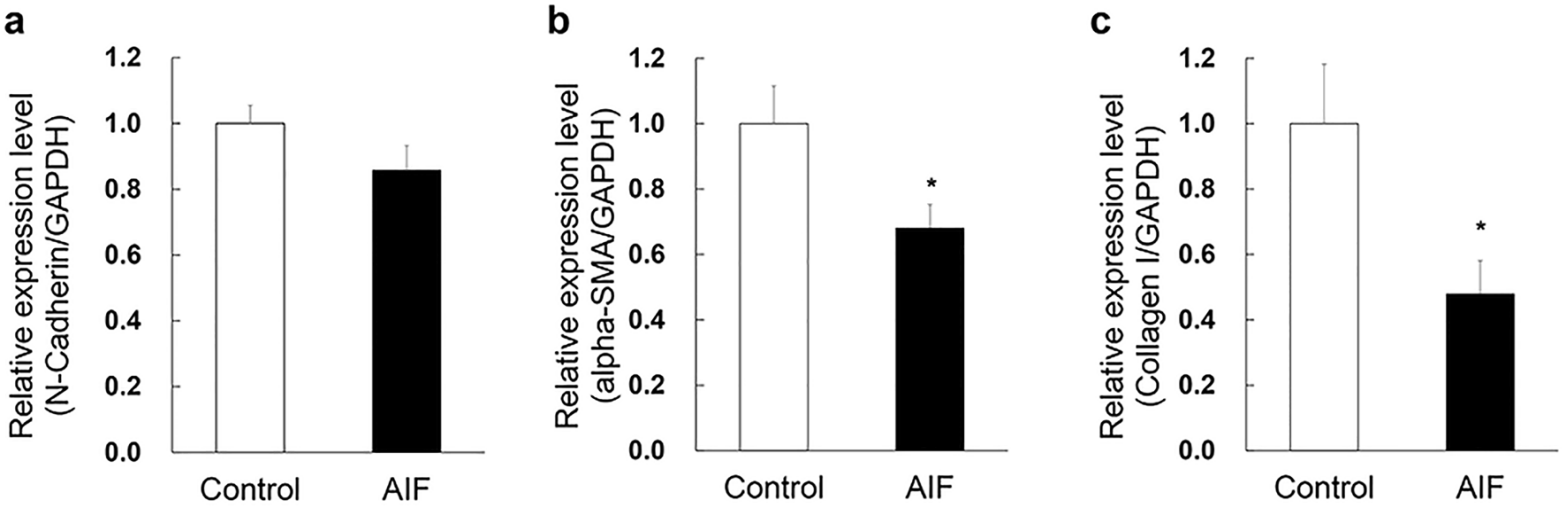
Inhibitory effects of alpinumisoflavone (AIF) on the expression fibrosis marker of ARPE-19 cells. ARPE-19 cells were treated with 5 µM AIF. AIF reduced the RNA expression level of (**b**) alpha smooth muscle actin (alpha-SMA) and (**c**) collagen I in ARPE-19 cell lysates. The statistical analysis was performed using Mann–Whitney U test (*p < 0.05 vs. control group) and the results are expressed as mean ± SEM (n = 3 biological replicates). qRT-PCR for each biological replicate was performed in duplicate.

We used qPCR to determine the expression levels of fibrosis markers (Fig. 3). We normalised the quantified mRNA of each gene to the corresponding glyceraldehyde 3-phosphate dehydrogenase (GAPDH) mRNA levels. We showed that AIF reduced the mRNA expression level of alpha-SMA and collagen I but not N-Cadherin in ARPE-19 cells (Fig. 3b; *p* = 0.0164; Fig. 3c; *p* = 0.0205).

### AIF and bevacizumab decreased the CNV area in laser-induced mouse model

**Figure 4:**
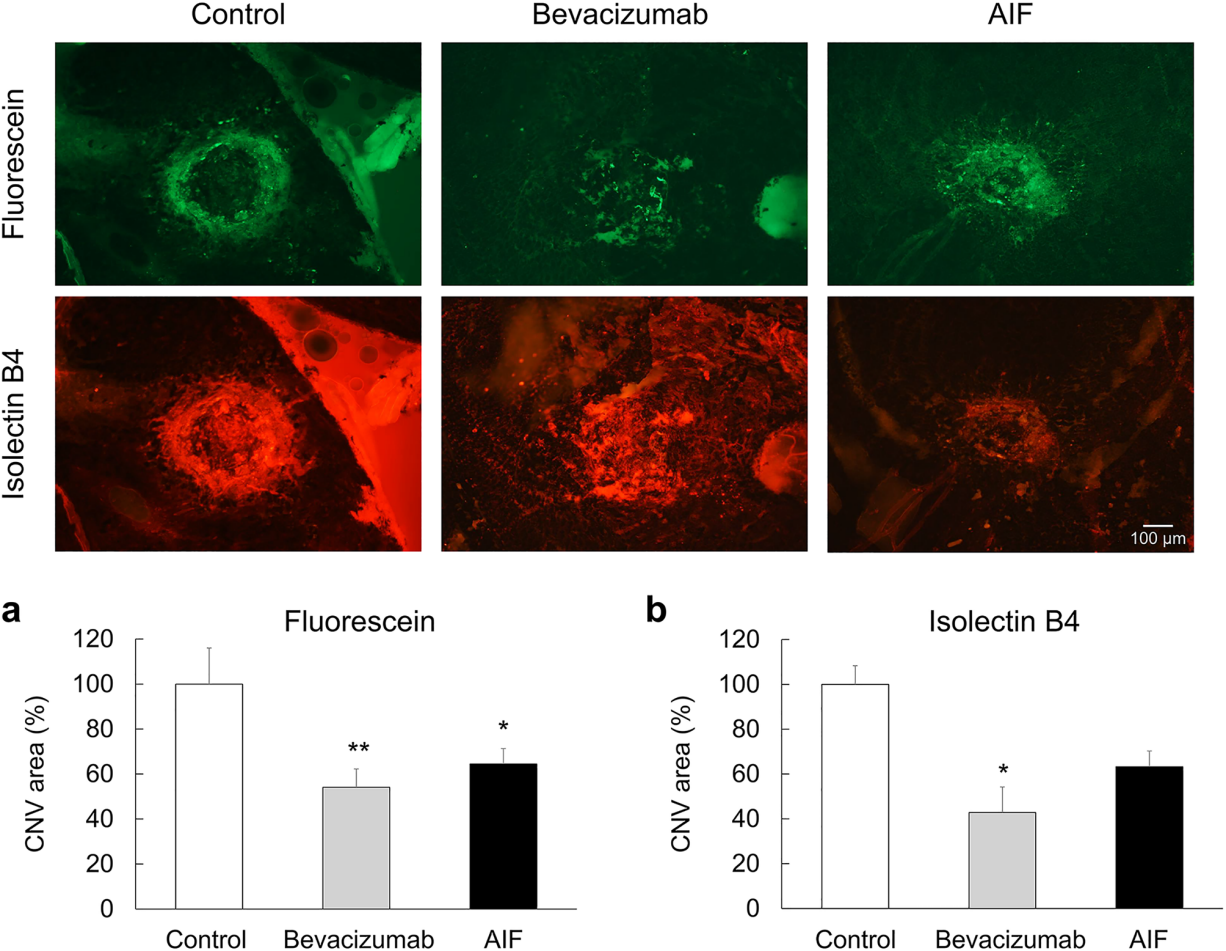
Effects of alpinumisoflavone (AIF) on laser-induced mouse model on choroidal neovascularisation (CNV) area. AIF suppresses the development of the CNV area in the laser-induced CNV mouse model. Representative images of CNV lesions and quantification of its area and volume in mice eyes that underwent laser photocoagulation administered with bevacizumab or AIF (Both 1 μL, 25 μg, intravitreal injection). One week later, the CNV area was analysed via measurement of fluorescence intensity of images with (**a**) fluorescein and (**b**) isolectin B4 positive area. Data were analysed using one-way ANOVA followed by the Holm-Šídák *post-hoc* t-test (*p < 0.05 and **p < 0.01 vs. control group). The results are expressed as mean ± S.E. (n = 7–9). The scale bar in each image is 100 μm.

To evaluate the effects of AIF on CNV development, we measured the CNV area in laser-induced mouse model. Immediately after laser photocoagulation, a single intravitreal injection of AIF was administered to the mice. Bevacizumab intravitreal injection was also administered as a positive control group. A week later, CNV lesions were visualised by perfusing mice with fluorescein and staining the choroidal vasculature with isolectin B4 (Fig. 4). AIF and bevacizumab-treated eyes had a reduction in the area of CNV lesions compared with that in the non-treated control eyes (Fig. 4a; *F*(2,22) = 10.105, *p* < 0.001; Fig. 4b; *F*(2,22) = 4.477, *p* = 0.023).

### AIF reduced the expression level of VEGFA and alpha-SMA in laser-induced CNV mouse model

**Figure 5:**
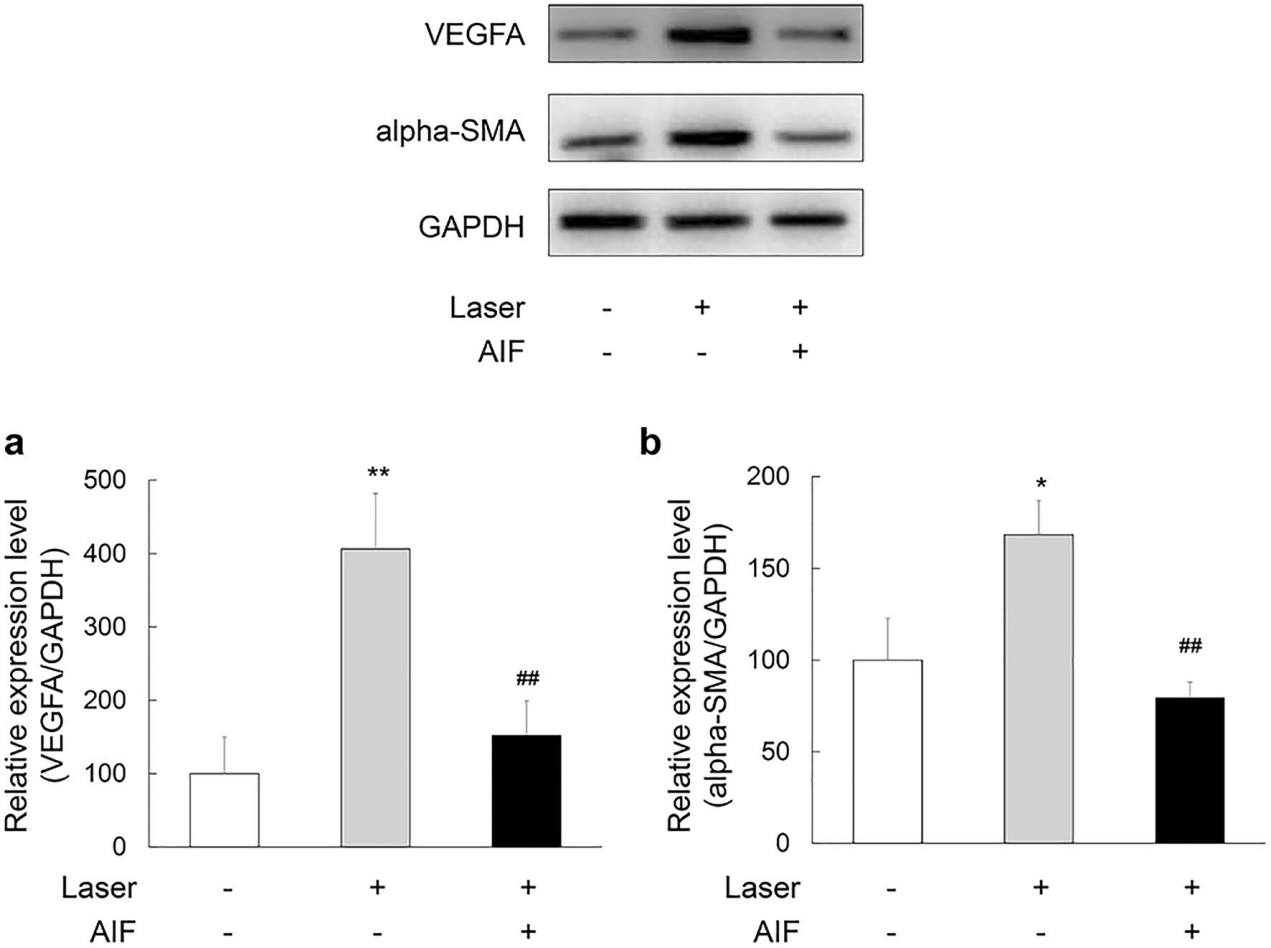
Inhibitory effects of alpinumisoflavone (AIF) on VEGFA and alpha smooth muscle actin (alpha-SMA) expression in the laser-induced choroidal neovascularisation (CNV) mouse model. Intravitreal injection of 25 mg/mL AIF was administered immediately after laser exposure. Equal amounts of protein from laser-exposed mice eye tissue lysates were analysed for expression of the indicated proteins. Protein expression in CNV of laser-exposed eyes of mice was measured a week after laser exposure by western blot analysis. Data were analysed using one-way ANOVA followed by the Holm-Šídák *post-hoc* t-test (*p < 0.05 and **p < 0.01 vs. non-laser exposed group, ^##^*p* < 0.01 vs. only Laser exposed group) and the results are expressed as mean ± S.E. (n = 4–5). Uncropped blot images are presented in supplementary information (Supplementary Fig. 3).

We performed a western blot assay to evaluate the effect of AIF on the expression of VEGFA and alpha-SMA in laser-induced CNV mice model. Seven days after laser photocoagulation, we removed the eyeballs of the experimental mice and extracted proteins. Western blot analysis of VEGFA and alpha-SMA was performed, with, GAPDH as an internal loading control. We found a decrease in the expression levels of VEGFA and alpha-SMA in the eyes treated with AIF compared to those in eyes treated without AIF (Fig. 5a; *F*(2,11) = 8.836, *p* = 0.005; Fig. 5b; *F*(2,11) = 8.108, *p* = 0.007).

### Morphological changes induced by laser photocoagulation

**Figure 6:**
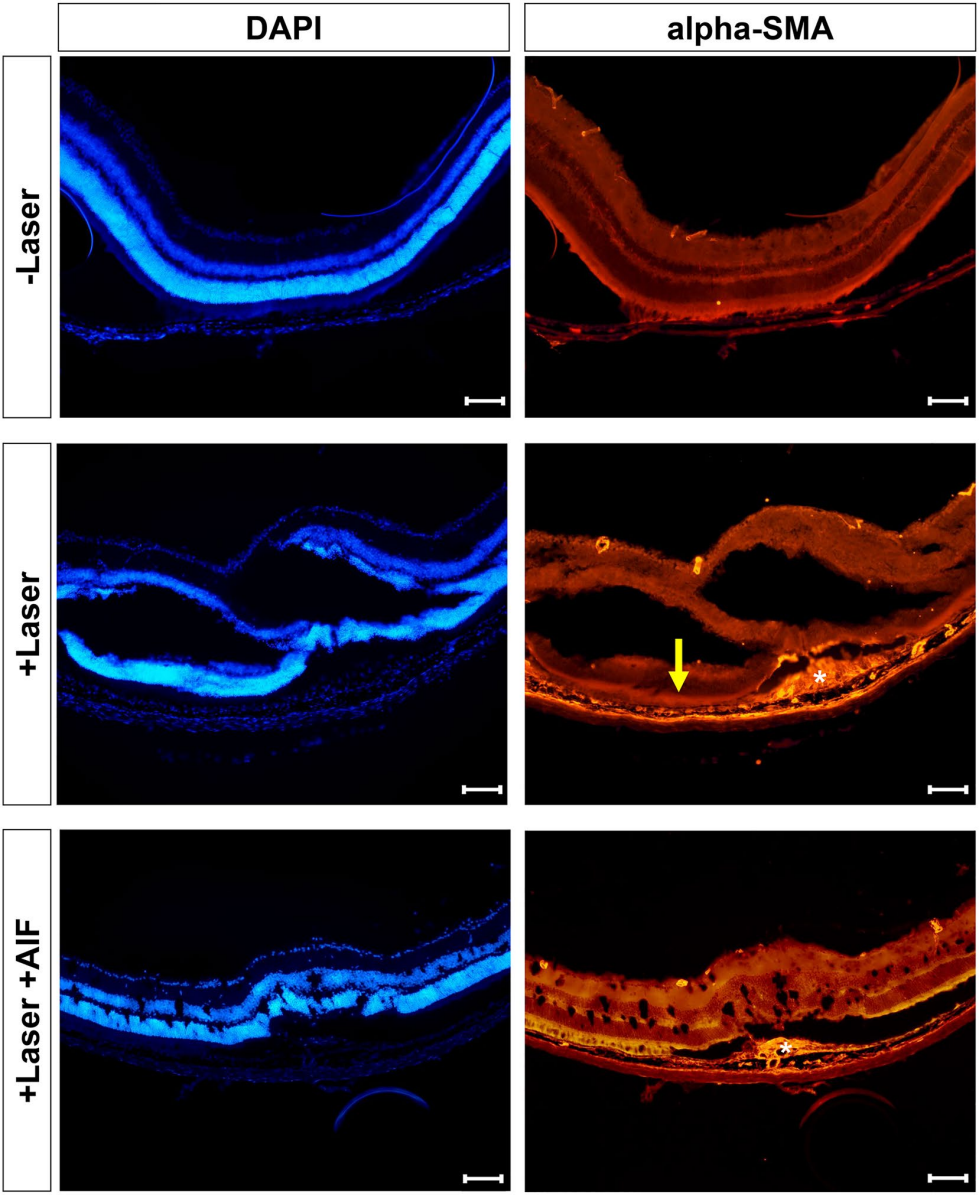
Expression of alpha smooth muscle actin (alpha-SMA) in laser-exposed mouse eye. Immunohistochemical staining of eyes from the non-laser induced mice and laser-induced CNV model with or without AIF treatment stained for DAPI (blue) and alpha-SMA (red). White asterisks indicate subretinal fibrosis and increased alpha-SMA expression level. Yellow arrow depicts retinal pigment epithelium (RPE) layer. The scale bar in each image is 100 μm.

To investigate the morphological changes in laser-induced mice eyes, the mice were euthanised 7 days after laser photocoagulation. Then, immunohistochemical analyses was performed. In mice eye sagittal sections, the subretinal infiltrates and fibrosis were observed in laser exposed subretinal lesions. The expression levels of alpha-SMA was increased 7 days after laser photocoagulation but was reduced in AIF treated mice eye section (Fig. 6).

### AIF reduced the cleaved form of CRYAB and phosphorylated form of CRYAB (p-CRYAB) expression in laser-induced CNV mouse model

**Figure 7:**
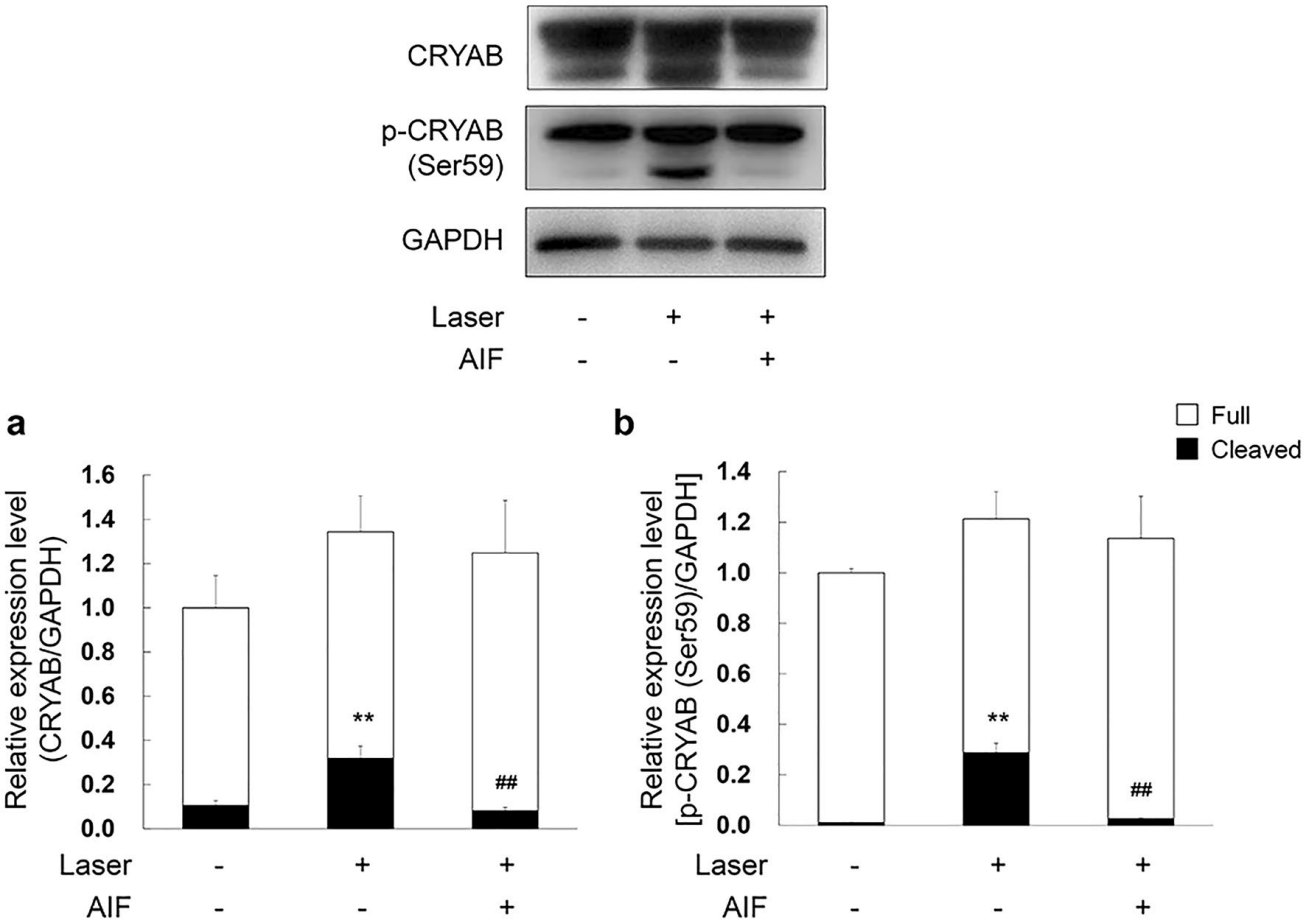
Inhibitory effects of alpinumisoflavone (AIF) on crystallin alpha B (CRYAB) and phosphorylated-CRYAB (p-CRYAB) expression in laser-induced choroidal neovascularisation (CNV) model. Intravitreal injection of 25 mg/mL AIF was administered immediately after laser exposure. The tissue lysates from three animals were pooled as one sample and equal amounts of protein were analysed for determining expression of the indicated proteins. The cleaved form of (**a**) CRYAB and (**b**) p-CRYAB reduced by 25 mg/mL AIF treatment in the laser-induced CNV model. Experiments were performed in triplicate and data were analysed using one-way ANOVA followed by the Holm-Šídák *post-hoc* t-test (**p < 0.01 vs. non-laser exposed group, ^##^*p* < 0.01 vs. only Laser exposed group). The results are expressed as mean ± S.E. (n = 3). Each error bars represent standard deviation from triplicate reading of three pooled samples. Uncropped blot images are presented in supplementary information (Supplementary Fig. 4).

It is known that CRYAB is regulated angiogenesis by modulation of VEGF^31^. Therefore, we analysed the expression of CRYAB and p-CRYAB in laser-induced CNV mice model using western blot assay. Seven days after laser photocoagulation, we removed the eyeballs of the experimental mice and extracted proteins. Western blot analysis of the cleaved form of CRYAB and p-CRYAB was performed, with GAPDH as the internal loading control. We found a decrease in the expression levels of the cleaved form of CRYAB and p-CRYAB in the eyes of mice treated with AIF compared to those in eyes treated without AIF (Fig. 7a; *F*(2,6) = 52.690, *p* < 0.001; Fig. 7b; *F*(2,6) = 14.026, *p* = 0.005.

### CRYAB interacted with alpha-SMA in the laser-induced CNV mouse model

**Figure 8:**
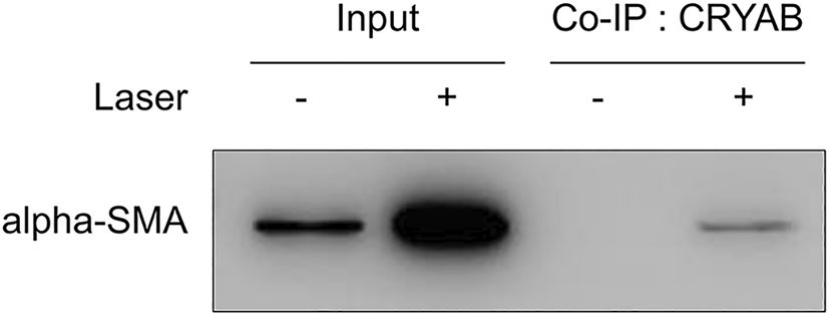
Interaction between crystallin alpha B (CRYAB) and alpha smooth muscle actin (alpha-SMA). Western blot analysis of proteins following the co-immunoprecipitation assay. Laser-exposed mice eye tissue lysates were co-immunoprecipitated with CRYAB. Alpha-SMA was successfully co-immunoprecipitated with CRYAB. Uncropped blot images are presented in supplementary information (Supplementary Fig. 5).

We performed Co-immunoprecipitation (Co-IP) assay to evaluate the association between CRYAB and alpha-SMA. A week after laser exposure, we removed the eyes of the experimental mice and extracted proteins. In Co-IP assays, input (whole cell lysate from mouse eye) was used as a control to determine whether IP was successful, and the results showed that CRYAB interacted with alpha-SMA in the laser-induced mouse model (Fig. 8).

## Discussion

nAMD is a leading cause of blindness with symptoms of neovascularisation, haemorrhage, inflammation, and fibrosis in the subretinal region of the elderly population. Anti-VEGF signalling antibody therapeutics, such as bevacizumab, ranibizumab, and aflibercept, are prescribed as primary medications for nAMD therapy to inhibit neovascularisation^4–7,32,33^. However, the development of subretinal fibrosis after anti-angiogenesis treatment is one of the major concerns in the prognosis of patients with nAMD. In a study conducted by Daniel et al., approximately 45% of patients developed fibrotic scarring 2 years after anti-VEGF treatment^8^. Thus, fibrotic scarring is a primary biomarker to predict loss of vision in patients with nAMD.

In this study, we examined the effects of AIF on VEGF expression and fibrosis in AMD models. We demonstrated that AIF significantly reduced released VEGFA in H_2_O_2_ treated ARPE-19 cells. Thus, these findings suggest that AIF may inhibit chorioretinal neovascularisation by reducing VEGF induced by oxidative stress. Parallel to previous studies, we found that AIF has an inhibitory effect on oxidative stress and angiogenesis^17,34^.

Furthermore, TCM induced the increase in VEGF expression in ARPE-19 cells. However, AIF co-treatment with TCM reduced VEGF expression. According to the previous reports, activated macrophages are related to AMD pathological process^24,25^. In addition, it has been reported that VEGF mediates angiogenesis and inflammation^35^. Activated immune cells, such as macrophages, are the key source of cytokines in the inflammatory responses, which were ameliorated by AIF^34^. We demonstrated that AIF may inhibit VEGF production and cellular response to activated macrophages. We take this as evidence that AIF may suppress the production of VEGF via the regulation of macrophage-induced pro-inflammatory mediators.

The mRNA and protein levels of a fibrosis marker, alpha-SMA, and collagen I also decreased upon AIF treatment of ARPE-19 cells. These results encourage the examination of the potential protective role that AIF plays in the nAMD animal model. We demonstrated that AIF treatment significantly reduced laser-induced choroidal neovascularisation in the mouse model. Inhibitory activity of AIF was similar to that of bevacizumab (25 μg/eye). Furthermore, AIF also reduced VEGFA and alpha-SMA expression in the laser-induced CNV animal model. These results suggest that AIF may ameliorate choroidal neovascularisation development and reduce fibrosis related marker, alpha-SMA^36^ in the nAMD animal model.

We identified that AIF modulates AMD pathophysiology via CRYAB function. We showed that CRYAB may act as a co-regulator of alpha-SMA, using Co-IP assay and gene–gene interaction analysis [genemania.org (Supplementary Fig. 1)]. According to previous studies^31,37^, CRYAB also interacts with and stabilises the VEGF structure, which plays an important role in the pathological process of AMD. These results suggest that CRYAB may bind to VEGF and alpha-SMA, and consequently act as a chaperone to enhance the function of target molecules in the subretinal fibrosis and CNV process. CRYAB has several phosphorylated sites, such as S19, S45, and S59. Increased VEGFA secretion and angiogenesis are associated with the upregulated S59-phosphorylated form of CRYAB (p-CRYAB)^31^.

It can be inferred from our study that AIF may be used independently because they have an effect similar to that of bevacizumab. We demonstrated that the binding between alpha-SMA and CRYAB increased in the nAMD animal model. In addition, AIF treatment reduced the cleaved form of CRYAB and p-CRYAB in the laser-induced CNV model. However, the expression levels of total CRYAB and total p-CRYAB were not affected by AIF treatment. Therefore, we suggest that AIF may decrease the cleavage of CRYAB and the transcription of alpha-SMA, as mentioned above. CRYAB is a heat shock protein that binds to VEGFA and consequently inhibits degradation and escalates the release of the molecule. AIF treatment may increase the degradation of VEGF via the inhibition of chaperone function in the nAMD model. Overall, we can conclude that AIF reduces fibrosis scarring as well as CNV development via the regulation of transcription of alpha-SMA, expression of the cleaved form of CRYAB, and VEGF function in the nAMD model.

## Methods

### AIF preparation

AIF was extracted from the unripe fruits of *Maclura tricuspidata* and purified as per previously reported protocols^38,39^. Briefly, methanol extract made from 556.0 g of unripe fruits and 100% methanol was suspended in distilled water and partitioned successively with n-hexane, methylene chloride, ethyl acetate, and n-butanol. The methylene chloride fraction, subjected to a Sephadex LH-20 column eluted with methanol, yielded four sub-fractions (CTUM1–CTUM4). AIF was purified from CTUM3 using semi-preparative high-performance liquid chromatography (HPLC) with acetonitrile/distilled water (57:43). The AIF (> 95% pure, as measured via HPLC analysis using a PDA detector) was dissolved in dimethyl sulfoxide (DMSO, Sigma-Aldrich, St. Louis, MO, USA).

### Cell culture

ARPE-19 cells (ATCC, Manassas, VA, USA) were maintained in Dulbecco's modified Eagle’s medium (DMEM, Gibco, Waltham, MA, USA), supplemented with 10% foetal bovine serum and antibiotic–antimycotic (100 units/mL, Gibco, Waltham, MA, USA), in a humidified incubator at 37 °C and 5% CO_2_. The media was changed thrice a week. For subculture, 1 mL of 0.5% trypsin–EDTA (Gibco, Waltham, MA, USA) was added and incubated at 37 °C for 3 min. Thereafter, 10 mL complete DMEM was added to harvest the cells via centrifugation at 3000 rpm for 3 min. The supernatant was then removed, and the pellet was resuspended in complete DMEM. Thereafter, the cells were seeded in 96-well plates at a density of 1 × 10^4^ cells/well.

### In vitro TCM and oxidative stress model

LPS-stimulated THP-1 cells were extensively used as a model of inflammation^40^. The human monocytic cell line THP-1 (Korean Cell Line Bank, Seoul, Korea, KCLB-40202) was cultured in RPMI-1640 (Hyclone, Logan, UT, USA) supplemented with 10% foetal bovine serum, 1% 2-mercaptoethanol (Gibco, Waltham, MA, USA), and antibiotic–antimycotic (100 units/mL, Gibco, Waltham, MA, USA), in a humidified incubator at 37 °C and 5% CO_2_. After reaching confluency, the cells were seeded at a density of 1 × 10^6^ cells/well in 12-well culture plates. For cell differentiation, THP-1 cells were treated with 200 ng/ml of PMA, Sigma-Aldrich, St. Louis, MO, USA) and incubated overnight at 37 °C and 5% CO_2_. Then, the PMA-supplemented media was removed, and the differentiated macrophage-like cells were stimulated with 1 μg/ml of LPS (Sigma-Aldrich, St. Louis, MO, USA) in RPMI-1640. After 24 h, the TCM was harvested, and the cells and debris were removed by centrifugation for 5 min at 3500 rpm. ARPE-19 cells were seeded in 96-well plates at a density of 1 × 10^4^ cells/well. On the second day, the ARPE-19 cells were treated with 0.1% DMSO in RPMI 1640 complete media or AIF (0, 1, 5, or 10 μM) in TCM for 3 h. Thereafter, culture media were harvested as mentioned above, and dot blot analysis was performed to detect VEGFA expression in culture media. TCM untreated ARPE-19 cells was used as a control. The fluorescence intensity of alpha-SMA and VEGFA was measured by the immunofluorescence analysis as described in the “Methods” section below.

Oxidative stress plays an important role in the pathogenesis of AMD^26–30^. According to previous studies^29,30^, we used H_2_O_2_ to induce VEGF production in ARPE-19 cells. Twenty-four hours after seeding, the ARPE-19 cells were treated with H_2_O_2_ or 5 μM AIF for 24 h and analysed via immunocytochemistry (ICC) as described in the “Methods” section below. Quantitative PCR (qPCR) was also performed as described in the “Methods” section below. After 24 h of H_2_O_2_ treatment, ARPE-19 cells were treated with 5 μM AIF for 24 h and analysed via dot blotting. In the above experiments, untreated cells were used as the control.

### Dot blot analysis

Cell media stimulated with ARPE-19 were centrifuged at 3500 rpm for 5 min. The supernatant was blotted onto nitrocellulose membrane by vacuum and Bio-Dot Apparatus (Bio-rad) and subsequently blocked in TBS containing 5% skim milk for 1 h. The membrane was then incubated with anti-VEGFA antibody (Abcam, ab46154, 1:1000) for 3 h at room temperature and washed thrice with TBST (5 min each). The membrane was then incubated with goat anti-rabbit IgG-HRP (Cell Signaling, 7074S, 1:5000) secondary antibody for 1 h at room temperature, washed again, and finally visualised using ECL substrate (Millipore, Billerica, MA) and a FUSION Solo S chemiluminescence detection system (Vilber Lourmat, Collégien, France).

### Immunocytochemistry/immunofluorescence (ICC/IF)

For immunocytochemistry, ARPE-19 cells were fixed in 4% paraformaldehyde for 20 min at room temperature and were permeabilised with 0.1% Triton X-100 in phosphate-buffered saline (PBS; Life Technologies, Carlsbad, CA, USA) for 15 min. Following blocking with PBS containing 5% goat serum and 0.1% Triton X-100 for 1 h at room temperature, fixed cells were incubated overnight at 4 °C with VEGFA (Santa Cruz Biotechnology, Santa Cruz, CA, USA, SC-7269, 1:300) in PBS containing 5% goat serum and 0.1% Triton X-100. Stained cells were washed with PBS containing 1% goat serum and 0.1% Tween 20. Then, the cells were incubated for 2 h at room temperature with the goat anti-rabbit IgG labelled with Alexa Fluor 488 (Abcam, ab150077, 1:500) or Goat Anti-Mouse IgG H&L (Alexa Fluor^®^ 568) (Abcam, ab175473, 1:500) in PBS containing 5% goat serum in 0.1% Triton X-100 in the dark. The cells were then stained with 4′,6-diamidino-2-phenylindole (DAPI, diluted to 300 ng/mL in PBS) for 10 min at room temperature in the dark. Cells were washed three times in PBS for 5 min each in the dark. Samples were stored in 0.02% (w/v) sodium azide in PBS. Thereafter, fluorescent images were captured with a Zeiss Axio Imager A2 microscope (Carl Zeiss, Oberkochen, Germany).

For immunofluorescence analysis, ARPE-19 cells were stained with alpha-SMA (Abcam, Cambridge, UK, ab5694, 1:300) and VEGF (Santa Cruz Biotechnology, Santa Cruz, CA, USA, SC-7269, 1:300) in the same way as above. Then, the fluorescence intensity was subsequently measured using a FlexStation 3 Multi-Mode Microplate Reader (Molecular Devices, LLC, Sunnyvale, CA, USA) as follows: DAPI, excitation wavelength of 340 nm and emission wavelength of 488 nm or alpha-SMA, excitation wavelength of 495 nm and emission wavelength of 519 nm or VEGFA, excitation wavelength of 578 nm and emission wavelength of 603 nm.

### Quantitative real-time PCR (qPCR)

RNA was extracted from ARPE-19 cells using QIAGEN RNA kit (QIAGEN, Hilden, Germany), and cDNA was synthesised from 1 μg total RNA using the High-Capacity cDNA Reverse Transcription Kit (Applied Biosystems, Foster City, CA, USA). Amplification reactions were performed with 12.5 μL 2 × SYBR Green PCR master mix (Applied Biosystems), 1 μg cDNA, and forward and reverse primers (100 μM each) using StepOne Plus Real-Time PCR System (Applied Biosystems). Cycling parameters were as follows: 50 °C for 2 min and 95 °C for 10 min; 1 cycle at 95 °C for 15 s and 40 cycles at 60 °C for 2 min, after one initial step at 95 °C for 30 s and a final step at 55 °C for 30 s. The primers used were as follows: alpha-SMA, forward 5′-AAAAGACAGCTACCTTGGGTGA-3′ and reverse 5′-GCCATGTTCTATCGGGTACTTC-3′; N-Cadherin, forward 5′-TCAGGCGTCTGTAGAGGCTT-3′ and reverse 5′-ATGCACATCCTTCGATAAGACTG-3′; Collagen I, forward 5′-TTGTGCGATGACGTGATCTGT-3′ and reverse 5′-TTGGTCGGTGGGTGACTCTG-3′; GAPDH, forward 5′-TGTCAAGCTCATTTCCTGGTATGA-3′ and reverse 5′-TCTTACTCCTTGGAGGCCATGTAG-3′.

### In vivo CNV model and drug treatment

Male wild-type C57BL/6N mice aged 8 weeks were randomly assigned to standard cages with 4–5 animals per cage (n = 5–8 for each group) and maintained under standard housing conditions in a 12 h light/dark cycle. For all procedures, animals were anaesthetised via intraperitoneal injection with ketamine (100 mg/kg body weight) and xylazine (10 mg/kg body weight). Pupils were dilated with a combination of 0.5% tropicamide and 0.5% phenylephrine (Mydrin-P; Santen, Osaka, Japan). Laser photocoagulation were performed in both eyes of each animal at the 3, 6, 9, and 12 o’clock positions of the retina, at a distance of 1–2 optic disc diameters surrounding the optic nerve, avoiding blood vessels. Laser spots were created with a spot diameter of 45 μm using the 78 diopters lens (VOLK, Mentor, OH, USA), 350 mW power, and 100 ms pulse duration using a laser indirect ophthalmoscope (OcuLight^®^ GL (Green 532 nm Laser), IRIDEX, Mountain View, CA, USA)^41^. Before intravitreal injection, a small amount of vitreous fluid was removed after puncture using a 30-g needle, being careful not to touch the lens on the limbus. Then, mice were intravitreally injected at the same puncture site with a 32-gauge needle attached to a 5 μL Hamilton microsyringe (Hamilton, Reno, NV, USA) as follows: 1 μL DMSO or AIF (25 μg in 1 μL DMSO) or 1 μL bevacizumab (Avastin, 25 mg/mL; Genentech, San Francisco, CA, USA).

### Fluorescein and Isolectin B4 staining of CNV lesions

Isolectin B4 staining was performed according to previously published reports but with a minor modification^42^. Mice were euthanised 7 days after laser photocoagulation. About 30 min before enucleation, 2.5% fluorescein sodium (Novartis, Basel, Switzerland) 0.5 mL was injected intraperitoneally. Residual fat, muscle, episcleral membrane, or optic nerve tissue was gently removed. The cornea and anterior sclera were dissected through an incision just anterior to the equator using microdissection scissors, and the lens, iris, and vitreous and neurosensory retina were removed. The planarization of the posterior scleral cup by making five meridian incisions starting at the equator and ending in the middle of the sclera approximately 1 mm from the centre of the optic nerve head did not damage the peripapillary sclera. Only RPE, choroid, and sclera were separated and placed in a 96 well plate. The samples were immediately fixed with 4% paraformaldehyde (Sigma-Aldrich, St. Louis, MO, USA) in PBS for 1 h at room temperature. For flat-mounts, posterior eye cups consisting of the RPE, choroid, and sclera were permeabilised with 0.1% Triton X-100 (Thermo Fisher Scientific, Waltham, MA, USA) in PBS for 1 h at room temperature. The CNV lesions were stained with Alexa Fluor 594-conjugated *Griffonia simplicifolia* isolectin B4 (1:100 dilution; Invitrogen, Carlsbad, CA, USA) and fluorescein at room temperature overnight. After three washes with PBS (15 min each), posterior eye cups were flat-mounted onto slides (Thermo Scientific) with the scleral side down in SlowFade anti-fade mounting medium (Life Technologies). Fluorescent images were captured using a Zeiss Axio Imager A2 microscope (Carl Zeiss).

### Immunohistochemical analyses

Mice were euthanised 7 days after laser photocoagulation. The eyes were post-fixed overnight with 4% PFA in PBS at 4 °C and then coronally cut with a Leica CM1850 microtome (15 µm thick sections). The sections were then permeabilised in PBS with 0.3% Triton X-100 and 0.3% hydrogen peroxide and blocked in 5% bovine serum albumin (BSA, with 1% goat serum) for 1 h at room temperature. The sections were incubated with primary antibodies diluted in 5% BSA at 4 °C overnight using rabbit alpha-SMA (Abcam, ab5694, 1:200). After rinsing three times in PBS, the slices were incubated with the corresponding Alexa Fluor-conjugated secondary antibodies diluted in 5% BSA for 2 h at RT and finally stained for 20 min with DAPI (300 nM in 5% BSA, Sigma-Aldrich). The sections were then mounted onto glass slides (Matsunami, Osaka, Japan) with mounting medium (Vector Laboratories, Inc., San Francisco, CA, USA). For each section, images were taken under a 5× objective using a Zeiss Axio Imager A2 microscope (Carl Zeiss).

### Western blot analysis

Mice retina, RPE, and choroid were isolated via sonication in lysis buffer (iNtRON Biotechnology, Seongnam, Korea) and centrifuged at 13,000 rpm for 25 min at 4 °C. The supernatant was obtained as total protein extracts. ARPE-19 cells were homogenised with lysis buffer (iNtRON Biotechnology) and centrifuged at 13,000 rpm for 25 min at 4 °C. The supernatant was obtained as total protein extracts. An equal amount of total protein was resolved on 12% sodium dodecyl sulfate–polyacrylamide gel followed by gel electrophoresis. The transferred PVDF membranes were incubated overnight at 4 °C with alpha-SMA (Abcam, ab5694, 1:1000), VEGFA (Abcam, ab46154, 1:1000) CRYAB (Abcam, ab13496, 1:2500), phospho-CRYAB (Ser59; Invitrogen, PA1-012, 1:2500), and GAPDH (Cell Signaling Technology, 2118S, 1:5000) antibodies. Thereafter, blots were incubated with corresponding conjugated goat anti-rabbit (Cell Signaling, 7074S, 1:5000) or horse anti-mouse IgG-HRP (Cell Signaling, 7076S, 1:5000) secondary antibodies. Immunoreactive proteins were detected with ECL substrate (Millipore) and visualised using a FUSION Solo S chemiluminescence detection system (Vilber Lourmat, Collégien, France).

### Co-immunoprecipitation (Co-IP) assay

Co-IP assays were performed using a Pierce Classic Magnetic IP/Co-IP Kit (Thermo Scientific) according to the manufacturer's instructions. Briefly, tissue lysate containing 500 μg total protein (quantified by bradford assay) was incubated with 2 μg CRYAB antibody for IP overnight at 4 °C. Thereafter, the antigen/antibody complex was incubated with 25 μL Pierce Protein A/G magnetic beads for 1 h at room temperature. The beads were then washed twice with IP Lysis/Wash Buffer and once with distilled water, and the antigen/antibody complex was subsequently eluted from the beads by heating at 100 ℃ for 5 min. Lastly, CRYAB was measured via western blotting.

### Statistical analysis

The data were analysed with Mann–Whitney U test using the Statistics Kingdom web calculator (https://www.statskingdom.com) and one-way analysis of variance (ANOVA) followed by Holm-Šídák *post-hoc* t-test with SigmaPlot 14 software (Systat Software, San Jose, CA, USA). The results are presented as mean ± standard error (S.E.).

### Ethical approval

Male wild-type C57BL/6N mice aged 8 weeks were handled in accordance with the Association for Research in Vision and Ophthalmology’s (ARVO) statement on the Use of Animals in Ophthalmic and Vision Research. This study was reviewed, and the protocol was approved by the Institutional Human Experimentation Committee Review Board of Ulsan University Hospital, Ulsan, Republic of Korea (NON2020-004) and Chungbuk National University, Cheongju, Republic of Korea (CBNUR-1453-20). All the animal experiments followed the ethical guidelines of ARRIVE (https://arriveguidelines.org).

## Supplementary Information

Supplementary Figures.

## Data Availability

The datasets used and/or analysed during the current study are available from the corresponding authors on reasonable request.
