# Supplementary material for: Alpinumisoflavone ameliorates choroidal neovascularisation and fibrosis in age-related macular degeneration in in vitro and in vivo models
Source: Sci Rep. 2022 Aug 22;12:14316. doi: 10.1038/s41598-022-18531-y (PMC9395367; doi:10.1038/s41598-022-18531-y)
Supplement: Supplementary file 1 — Supplementary Figures. [file 41598_2022_18531_MOESM1_ESM.pdf]

Supplementary information

Alpinumisoflavone ameliorates choroidal neovascularization and fibrosis in age-related macular degen eration *in vitro* and *in vivo* models

Eunhye Yu [1†], Yunjeong Song [1†], Sun Mi Gu[1], Yang Hee Jo[2], Sang Won Yeon[2], Kyu Jin Han[3], Mi Kyeong Lee[2], Jung Kee Min [1,3*], Jaesuk Yun [1*]

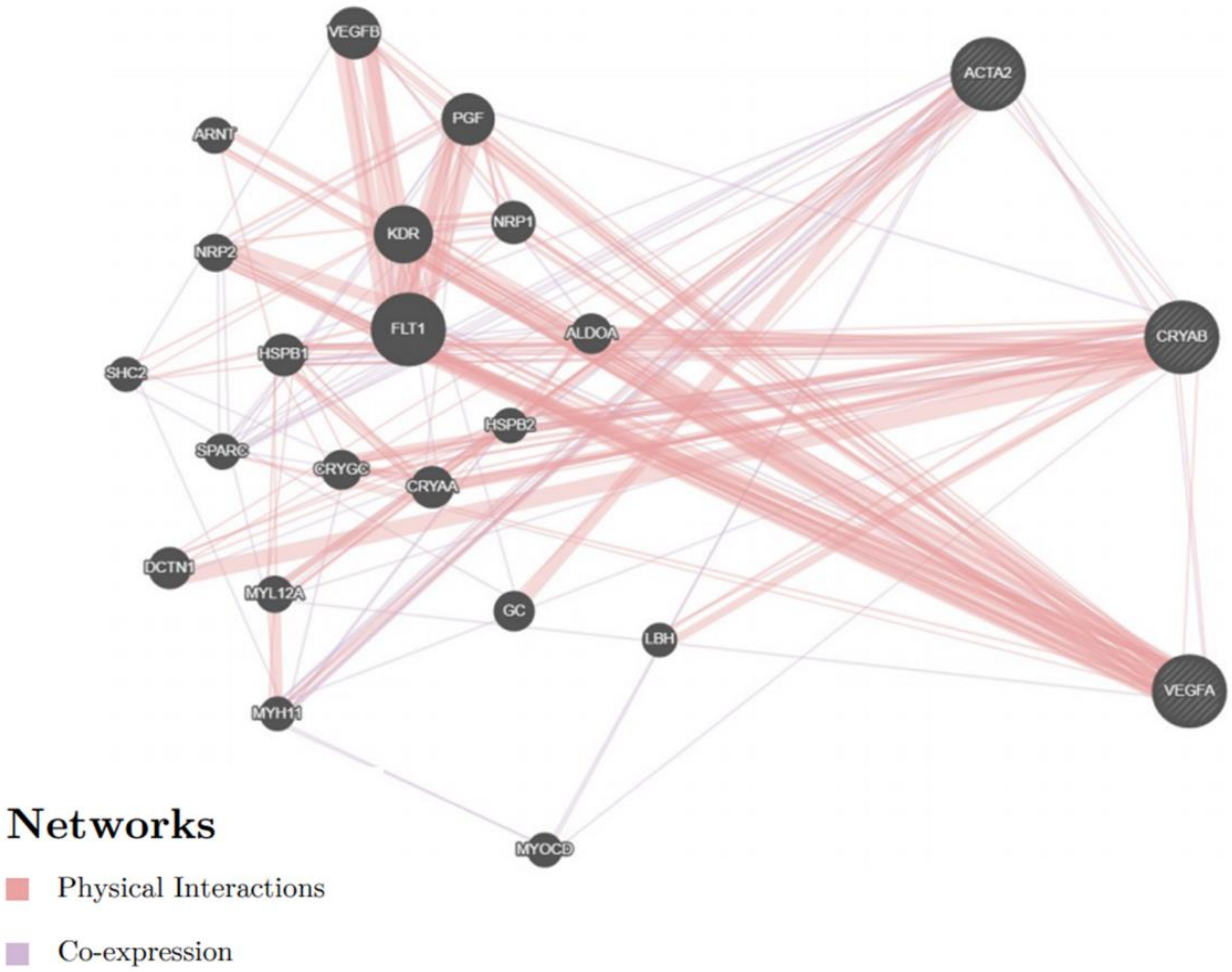

Supplementary Fig. 1. The gene-gene interaction network was generated by GeneMANIA (Warde-Farley et al., 2010). Color of lines represents the type of interaction in the network (left panel); physical interaction (pink), co-expression (purple).

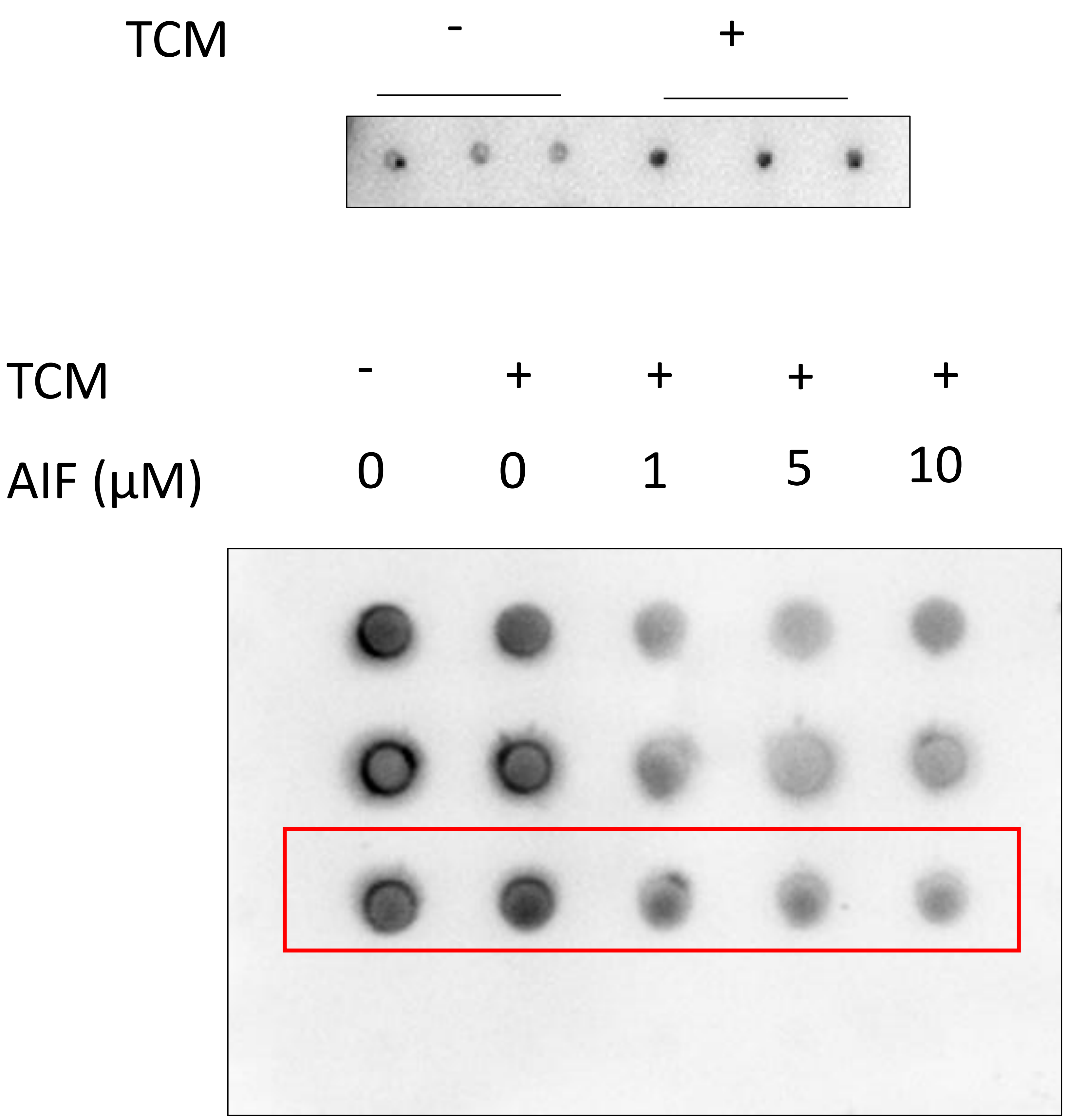

Supplementary Fig. 2. Original images of dot blots before cropping. Expression levels of VEGFA were assayed with western blot, respectively. The raw blots images of full length membranes and the red frames were the image present of Fig. 1.

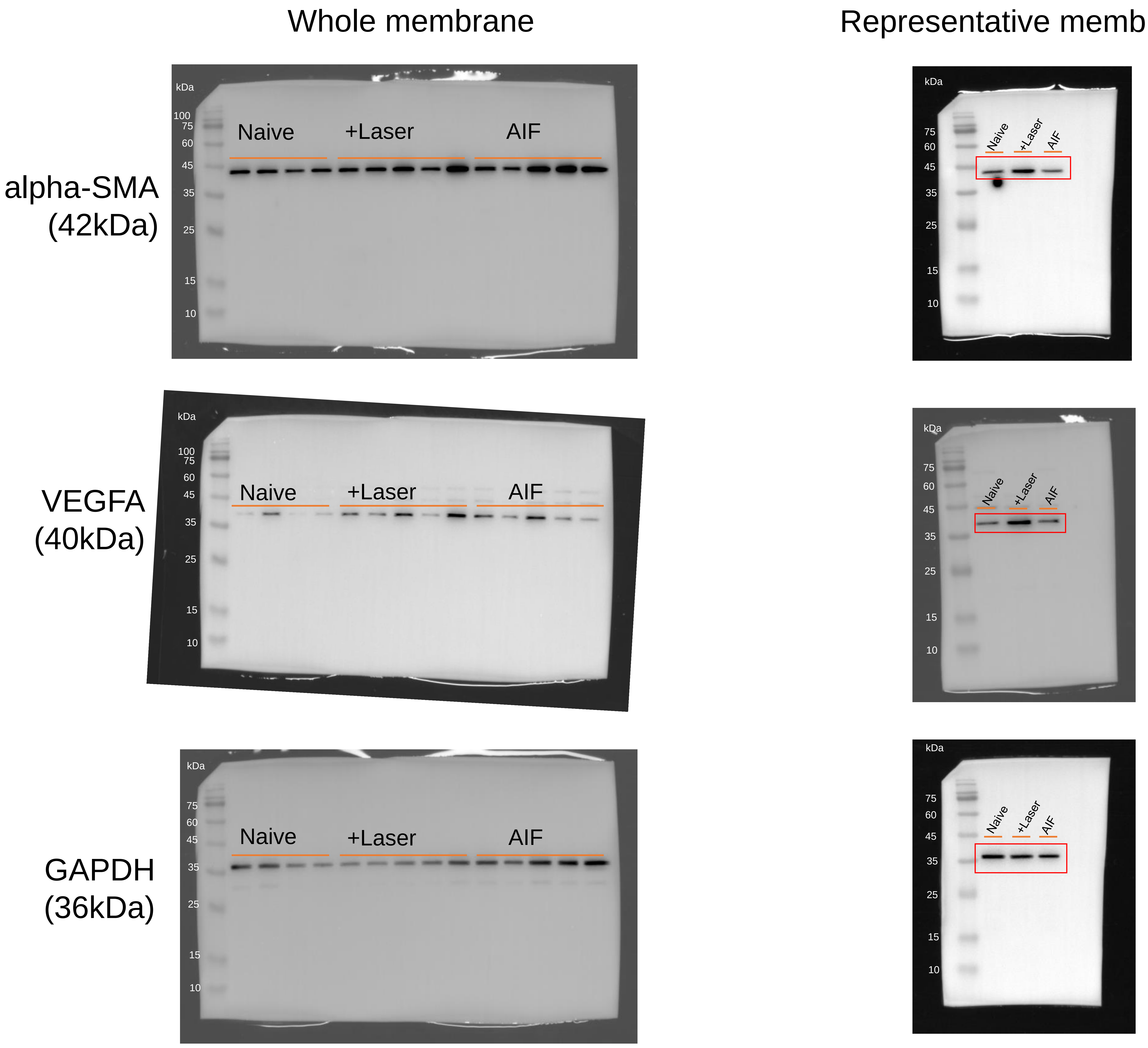

Supplementary Fig. 3. Original images of western blots before cropping. Expression levels of VEGFA and alpha-SMA were assayed with western blot, respectively. GAPDH was used as loading internal control. The raw blots images of full length membranes and the red frames were the image present of Fig. 5. All gels are 12%.

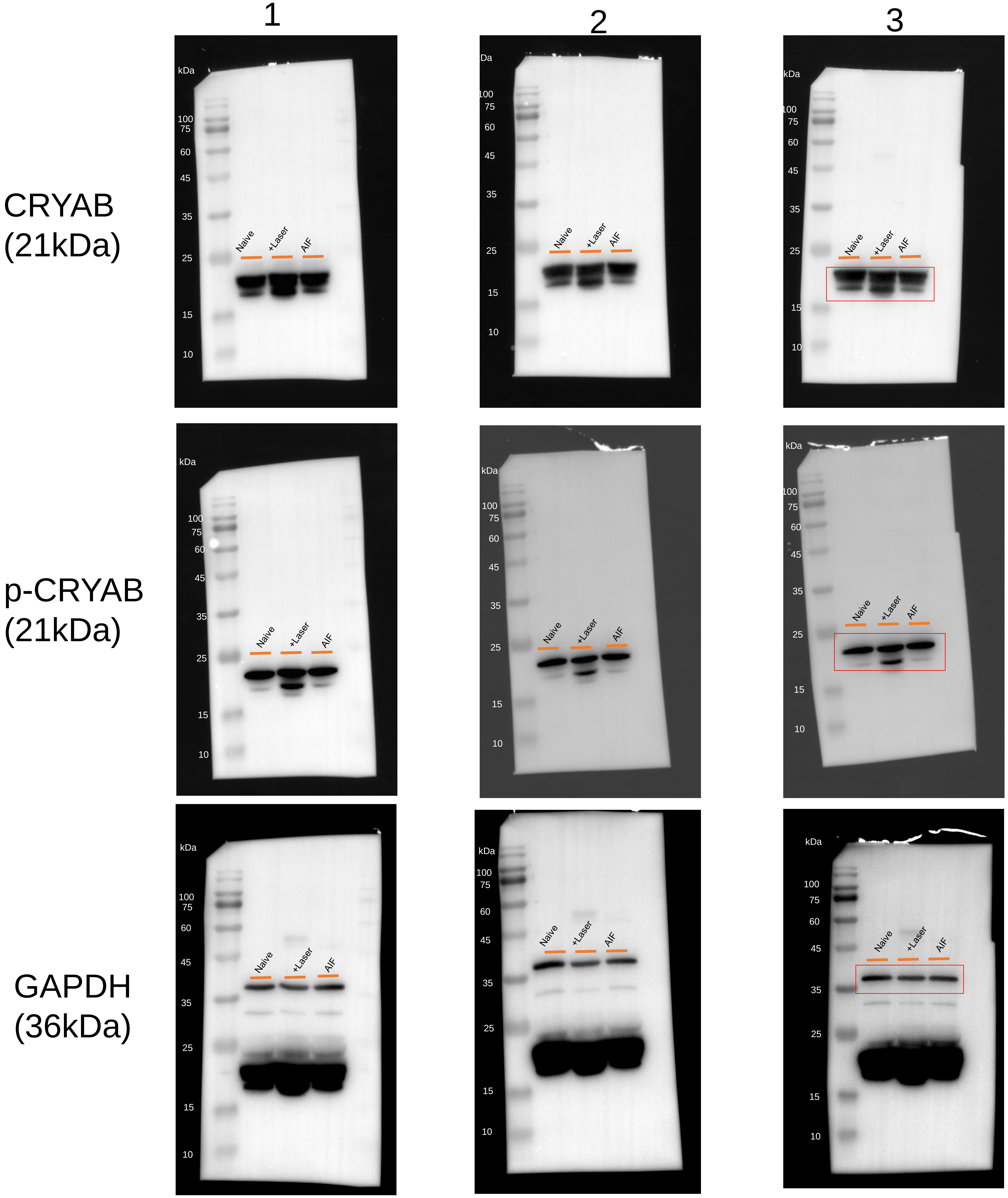

Supplementary Fig. 4. Original images of western blots before cropping. Expression levels of CRYAB and p-CRYAB were assayed with western blot, respectively. GAPDH was used as loading internal control. The raw blots images of full length membranes and the red frames were the image present of Fig. 7. All gels are 12%.

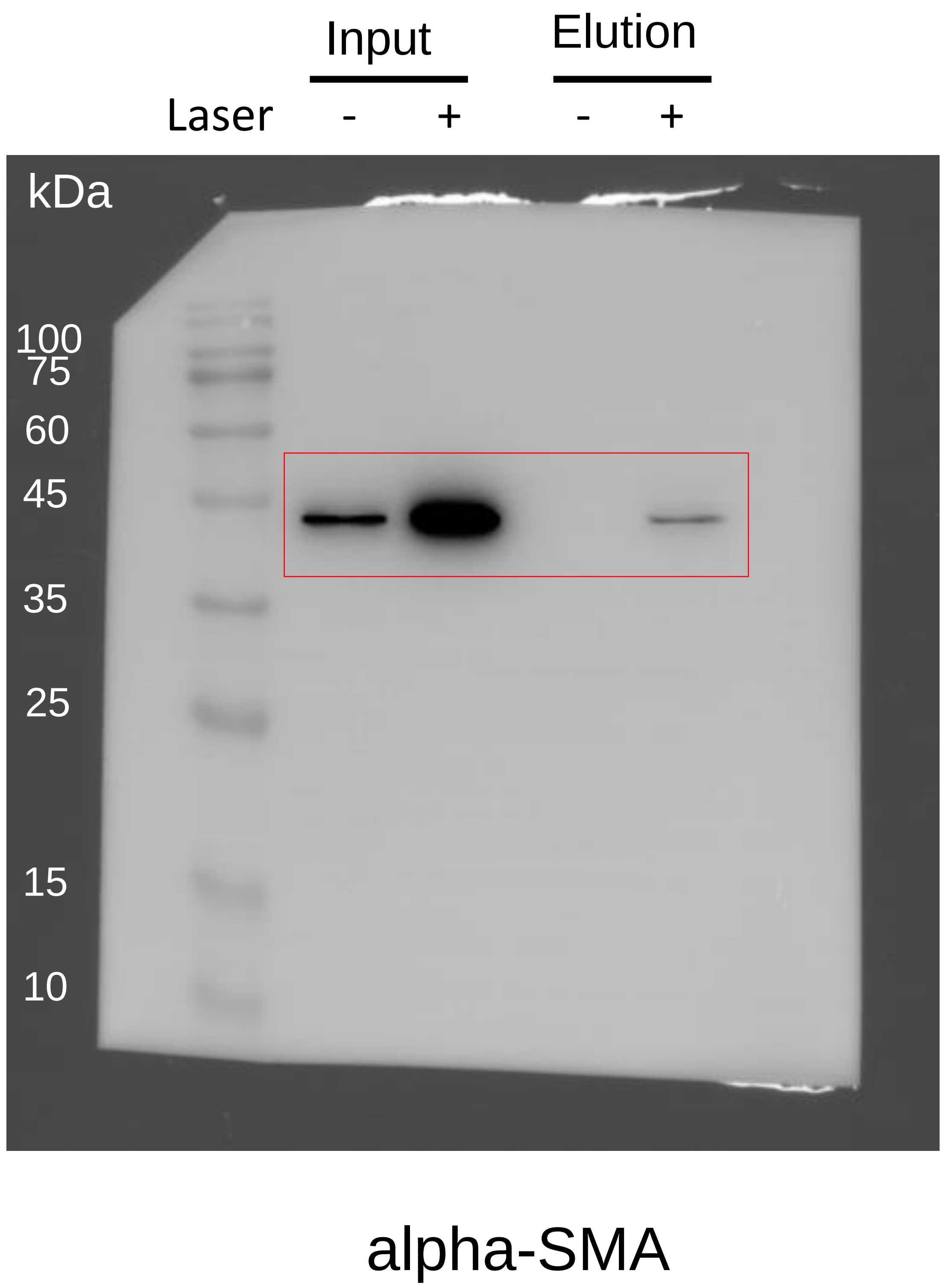

Supplementary Fig. 5. Original images of western blots before cropping. Full-length blot and a red frame was the image present of Fig. 8. A gel is 12%.
